# Transitioning Between Treprostinil Formulations: Evidence and Strategies

**DOI:** 10.1007/s12325-025-03401-6

**Published:** 2025-11-10

**Authors:** Megan Clarke, Ali Ataya, Alison M. Turkin, Raj Parikh, Kyle Davis, Chad E. Miller, Alexander Kantorovich, Thomas Winkler, Steven J. Cassady

**Affiliations:** 1 Novant Health New Hanover Regional Medical Center, Wilmington, NC USA; 2 University of Florida, Gainesville, FL USA; 3 United Therapeutics, Research Triangle Park, NC USA; 4 Hartford Hospital, Hartford, CT USA; 5 Piedmont Healthcare, Atlanta, GA USA; 6 University of Maryland School of Medicine, Baltimore, MD USA

**Keywords:** Treprostinil, Transitions, Route of administration, Pharmacokinetics, Pulmonary hypertension, Prostacyclin pathway-targeted therapy

## Abstract

Treprostinil is a prostacyclin analog available in four formulations: parenteral, oral, inhalation solution, and inhalation powder. All formulations of treprostinil are indicated for the treatment of pulmonary arterial hypertension; the inhaled formulations are also approved for pulmonary hypertension associated with interstitial lung disease. Transitions between formulations are frequent in clinical practice but lack standardized guidance. This review summarizes published evidence regarding safety, efficacy, and techniques of transitioning between treprostinil formulations. The evidence includes over 100 patients across various settings, with crossover transition strategies most frequently employed. Approximately 80% of cases reviewed were deemed successful; however, it should be noted there was no standardized definition for success across publications. Unsuccessful transitions were often linked to disease progression or intolerance to therapy. These findings underscore the importance of individualized transition strategies, informed by clinical status, patient preferences, and formulation-specific pharmacokinetics. By consolidating the current evidence, this review aims to support clinicians in optimizing transitions and maintaining continuity of treprostinil therapy.

Graphical abstract available for this article.

**Graphical Abstract:**

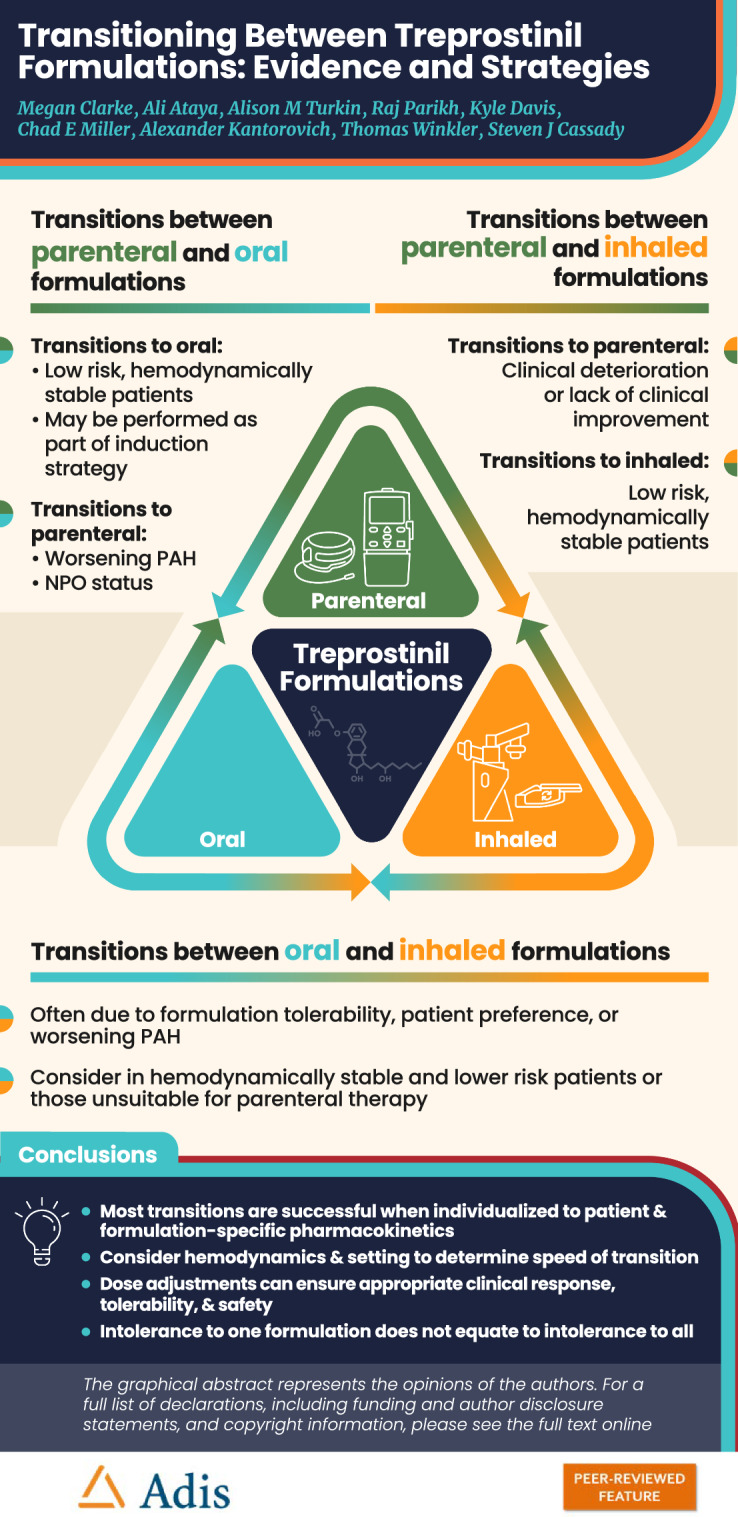

## Key Summary Points

**Table:** 

Transitions between formulations are frequently required in clinical practice due to changes in clinical status, adverse effects, administration challenges, or patient preference.
Patient-specific hemodynamics and transition setting should be considered to determine the speed of transition.
Dose adjustment is important to elicit the appropriate clinical response.
Most transitions were successful when individualized to patient needs and formulation-specific pharmacokinetics.
Intolerance to one formulation of treprostinil does not equate to intolerance to all formulations.

## Digital Features

This article is published with digital features, including a graphical abstract, to facilitate understanding of the article. To view digital features for this article, go to 10.6084/m9.figshare.30272929

## Introduction

Pulmonary hypertension (PH) is a pathophysiological condition described by an abnormal elevation in pulmonary artery pressure. Pulmonary arterial hypertension (PAH) is characterized by pulmonary vascular remodeling and vasoconstriction which can ultimately lead to right heart failure [1]. Prostacyclins, including treprostinil, are one of the four foundational pathways for the treatment of PAH. Treprostinil is available in parenteral, oral, inhalation solution, and inhalation powder formulations for the treatment of PAH. The inhaled formulations are also indicated for the treatment of pulmonary hypertension associated with interstitial lung disease. Treprostinil is a potent pulmonary vasodilator with anti-inflammatory, antifibrotic, and anti-proliferative effects and also inhibits platelet aggregation. Treprostinil is a chemically stable prostacyclin analog that acts as a full prostacyclin receptor (IP) agonist with a high affinity for the prostaglandin E receptor 2 (EP_2_) and the prostaglandin D receptor 1 (DP_1_). Stimulation of IP results in the activation of adenylate cyclase and the conversion of adenosine triphosphate to cyclic adenosine monophosphate. Activation of IP, EP_2_, and DP_1_ induces a G-protein-coupled cascade, yielding an increase in protein kinase A which mediates the therapeutic effects of treprostinil [2].

With the increasing armamentarium of PAH therapies, and recommendations to frequently reassess and escalate treatment early, transitioning between treprostinil formulations may often be warranted [3]. Transitions between formulations may also occur frequently in clinical practice due to tolerability, adverse effects, change in clinical status, patient preference, and pregnancy. The use of treprostinil requires careful consideration of each formulation’s pharmacokinetic properties, intended use, and advantages and limitations. If patients respond well to one formulation of treprostinil, demonstrating they tolerate prostacyclin-class adverse events, it is reasonable to maintain therapy within the prostacyclin class and transition between formulations based on individual need. At present, transitions between formulations occur without a guideline or consensus on the management and methods to perform such transitions. The aim of this review is to summarize published literature describing transitions between treprostinil formulations, and to serve as a resource to clinicians who can apply such data to their clinical practice.

## Treprostinil Formulations

### Parenteral

Current guidelines recommend parenteral treprostinil in high- and intermediate–high-risk patients with PAH as part of combination therapy [3, 4]. Parenteral treprostinil has been associated with significant reductions in right ventricular afterload and improvement in right ventricular function [5]. Parenteral treprostinil has been shown to improve clinical outcomes and may improve survival, particularly when the dose has been optimized [6, 7].

Parenteral treprostinil offers the advantages of consistent drug delivery and ease of dose titrations. Administration requires management of a pump to deliver treprostinil as a continuous infusion. Line-related bloodstream infections and intolerable injection site pain are common reasons for transition from parenteral treprostinil to other formulations. Patients may also be unable to effectively operate the pump due to limitations in dexterity or cognition, or may find the lifestyle limitations associated with pump management intolerable, prompting consideration for oral or inhaled treprostinil as alternative treatment options. Transitions may also be performed as part of an expedited approach to initiating patients on oral treprostinil, whereby a short course of parenteral treprostinil is administered while oral treprostinil is simultaneously up-titrated. Lastly, with the introduction of activin signaling inhibitors, patients may be eligible for transition from parenteral to oral or inhaled treprostinil due to significant clinical improvement and concurrent reduction in clinical risk scores.

### Oral

Oral treprostinil is approved for the treatment of PAH, where it has been shown to delay disease progression and improve exercise capacity [12]. Current guidelines recommend the addition of oral treprostinil in patients on background dual combination therapy with an endothelin receptor antagonist and phosphodiesterase-5 inhibitor who are at intermediate–low risk [3, 4]. Oral treprostinil is initiated at 0.125 mg (mg) three times daily (TID) or 0.25 mg twice daily (BID) and titrated not more frequently than every 3–4 days [13]. TID administration of oral treprostinil results in sustained plasma concentrations and similar exposure to parenteral treprostinil [14].

Similar to parenteral and inhaled prostacyclin, oral treprostinil has a dose-dependent response. Larger doses correlate with improvements in 6-min walk distance (6MWD) and a longer time to hospitalization [15]. Oral treprostinil can be started de novo as an addition to dual combination therapy, as a maintenance agent after induction therapy with parenteral treprostinil, or as a step-down agent in stable patients transitioning from parenteral treprostinil, depending on a patient’s risk status and clinical needs. A parenteral treprostinil dose of 6 ng/kg/min is equivalent to an oral dose of approximately 1 mg TID in a 70-kg patient [14].

### Inhaled

Inhaled treprostinil is available as a nebulized solution and as a dry powder inhaler (DPI). The inhaled route allows treprostinil to be delivered more directly to the site of action, which reduces systemic exposure and improves tolerability [11]. Inhaled treprostinil has been shown to improve exercise capacity and is currently recommended in patients at intermediate–low risk in addition to dual background therapy in patients with PAH [3, 4, 16]. The nebulized solution requires administration through a product-specific nebulizer device, which may prove cumbersome. The dry powder formulation was subsequently introduced as a more convenient option. The pre-filled cartridges and smaller, no-maintenance device may be preferred for patients leading active lifestyles. In one study, patients achieved higher doses with the dry powder compared to the nebulized formulation, enabling greater improvements in efficacy [17].

Both formulations are administered four times daily (QID) [18, 19]. There is not a prescribed formula, but several case reports and case series have proposed 9 breaths (54 µg) QID of nebulized treprostinil equates to approximately 10 ng/kg/min of parenteral treprostinil or 5 mg total daily dose of oral treprostinil [20].

## Tolerability

Adverse events associated with treprostinil are often classified as either prostacyclin class-related or formulation specific. Flushing, jaw pain, headache and diarrhea are generally considered class-related adverse events. In pivotal studies, infusion site pain and infusion site reactions were the most common adverse events associated with parenteral treprostinil, followed by headache, diarrhea, nausea, rash, and jaw pain [21]. With the exception of site pain, the severity and frequency of adverse events is often dose-dependent, and the rate of infusion and duration and speed of the titration are important considerations when adjusting for side effects. Site pain management should be personalized to each patient and generally improves after several months of therapy with effective pain-mitigation techniques [22, 23]. As with parenteral treprostinil, upward dose titration of oral treprostinil is often limited by class-related adverse effects, specifically myalgias, flushing, nausea, and diarrhea. As such, the inability to tolerate these effects is one of the most common reasons for transition from oral treprostinil.

In landmark trials involving inhaled treprostinil, cough was the most frequently mentioned adverse effect [16, 24, 25]. After cough, the most common adverse events associated with treprostinil inhalation solution are headache, throat irritation, and pharyngolaryngeal pain and headache, and dyspnea for treprostinil inhalation powder. Across all transitions, intolerance to side effects is the most frequently cited reason for transition.

## Methods of Review

A literature search was performed to identify publications reporting transitions between treprostinil formulations in patients receiving treatment for PAH between January 2003 and August 2024. Products included Remodulin^®^ (treprostinil) injection, Tyvaso^®^ (treprostinil) inhalation solution, and Orenitram^®^ (treprostinil) extended-release tablets, and Tyvaso DPI^®^ (treprostinil) inhalation powder. The search terms were: treprostinil injection, treprostinil inhalation solution, oral treprostinil, treprostinil inhalation powder, Remodulin, Tyvaso, Orenitram, Tyvaso DPI, transitions, transition strategy, treprostinil pharmacokinetics, and treprostinil formulations. Published materials included manuscripts, congress materials, and specialty pharmacy data. Publications reporting pediatric data or transitions to multiple agents without differentiating outcomes were excluded. This article is based on previously conducted studies and does not contain any new studies with human participants or animals performed by any of the authors.

## Transitions to Parenteral Treprostinil

### Oral to Parenteral Treprostinil

Few studies exist describing the transition from oral to parenteral treprostinil. These data consist of 2 case reports and 1 case series, totaling a sample of 12 patients [20, 26, 27]. Most patients were transitioned due to worsening PAH. All of the described transitions took place in the inpatient setting using a crossover technique, down-titrating oral treprostinil while simultaneously titrating up the parenteral treprostinil dose. Case reports by Pan and colleagues as well as Sargent et al. provide detailed descriptions of the transition process [20, 26]. Transitions took place over approximately 1 week with doses ranging from 17 to 90 ng/kg/min. All transitions were deemed successful with patients experiencing improvements in several clinical measures, such as World Health Organization (WHO) functional class (FC), hemodynamics, and echocardiography. Interestingly, 3 patients who were initially transitioned to parenteral treprostinil due to abdominal conditions that required perioperative nothing by mouth status were eventually transitioned back to oral treprostinil without complication following their surgery [27].

### Inhaled to Parenteral Treprostinil

One case report and one retrospective cohort study of patients transitioned from treprostinil inhalation solution to parenteral treprostinil were identified [28, 29]. Preston and colleagues performed a multicenter retrospective chart review of patients with WSPH Groups 1, 4, and 5 pulmonary hypertension undergoing transition from inhaled to parenteral treprostinil. A total of 26 patients were included, of which the majority had Group 1 PH, were WHO FC III, and were receiving background PAH therapy with at least one agent. Prior to transition, patients were treated with inhaled treprostinil for an average of 12.7 (range 2–50) months; 23/26 were receiving a dose of 9 inhalations QID or greater. The most common reason for transition was clinical deterioration (*n* = 19), followed by lack of clinical improvement (*n* = 6) and pregnancy (*n* = 1). Most transitions occurred in the inpatient setting (*n* = 17); however, transitions also occurred in the outpatient clinic (*n* = 7) and at home (*n* = 2). Most patients had their inhaled treprostinil discontinued with parenteral treprostinil initiated 6 h after the last dose of inhaled treprostinil and further up-titrated. A second cohort of patients had their inhaled treprostinil weaned off over several days, with parenteral treprostinil then initiated following the last dose of inhaled treprostinil (3 inhalations). Finally, a third cohort of patients underwent a crossover transition in which inhaled treprostinil was down-titrated while parenteral treprostinil was simultaneously titrated. Inhaled treprostinil was then discontinued when the parenteral dose approached 10–20 ng/kg/min. All transitions were successful, and no serious drug or hemodynamic effects occurred during or because of the transition. The most common side effects stemmed from prostacyclin excess and were addressed via dose adjustments. Follow-up ranged from 3–18 months, with most patients stabilizing or improving clinically [29].

## Transitions to Oral Treprostinil

### Parenteral to Oral Treprostinil

Several retrospective analyses and case reports exist describing the transition from parenteral to oral treprostinil in patients with PAH (Table 1) [30–40]. Infusion site pain, patient preference, intolerance to parenteral therapy, difficulty of administration, and recurrent infections were the most common reasons for transitioning to oral therapy. Several studies noted that only patients receiving stable doses of parenteral treprostinil that demonstrated clinical improvement while on therapy were felt to be eligible for transition. Doses of parenteral treprostinil prior to transition ranged from 15 to over 100 ng/kg/min, with duration of therapy ranging from less than 1 week to over 10 years. Most transitions occurred in the inpatient setting and utilized a cross-titration strategy, simultaneously decreasing the dose of parenteral treprostinil and increasing the dose of oral treprostinil over a period of several days. Most transitions were successful, and patients were maintained on oral therapy without hospital readmission or treatment-limiting adverse effects. Unsuccessful transitions often involved an increase in nausea and diarrhea or clinical deterioration, resulting in the transition back to parenteral therapy [30–40].

**Table 1:** Transitions from parenteral to oral treprostinil

Author, year (Reference)	Study design, sample size	NYHA FC, Background therapy	Dose prior to transition, duration of therapy	Setting of transition	Transition method, duration	Post transition oral TRE dose	Outcome
Balasubramanian (2018) [40]	Retrospective pharmacy claims analysis, *n* = 393	Not reported	44 ng/kg/min, 20.9 months	Not reported	Transition data NP	12 mg TDD (TID dosing) 7 mg TDD (BID dosing)	
Chakinala (2017) [14]	Prospective cohort study, *n* = 33	FC: I–II, ERA: 3 PDE5-I: 21 ERA + PDE5-I: 9	57 (25–111) ng/kg/min, not reported	Inpatient	Patients supervised for < 5 days in hospital and given oral TRE at roughly same time parenteral TRE was decreased During first 48 h parenteral TRE not decreased by more than 30 ng/kg/min Q24H and oral TRE not increased by more than TDD of 6 mg Q24H	Week 1: 33 (15.6–57.9) mg TDD	31/33 Transitions successful 6MWD and hemodynamic parameters were similar at 24-week follow-up 1 failed transition due to AEs 1 failed transition due to clinical worsening
Miller (2023) [41] Kingery (2023) [42]	Prospective cohort study, *n* = 29	FC: II–III, ERA: 1 PDE5-I or sGCS: 10 ERA + PDE5-I or sGCS: 12 None: 6	27 ± 9.6 ng/kg/min, 55 ± 13 days	Inpatient (*n* = 13) Outpatient (*n* = 16)	Patients initiated on parenteral TRE with a target dose of 20 ng/kg/min Oral TRE was initiated and cross-titrated at week 2,4, or 8 Mean inpatient transition length: 1.7 ± 0.5 days Mean outpatient transition length: 5.6 ± 2.3 days	16.6 mg ± 7.5 mg TDD	28/29 Transitions successful at 16 weeks Improvements noted in 6MWD, echocardiography, NT pro-BNP, and risk scores 1 patient experienced significant worsening of PAH and death unrelated to study drug
Maestas (2018) [39]	Retrospective cohort study, *n* = 24	FC: I–II, ERA: 2 PDE5-I: 13 ERA + PDE5-I:9	Successful transition group: 50 (27–70)ng/kg/min Unsuccessful transition group: 59 (38–84), not reported	Inpatient	Patients supervised for < 5 days in hospital and given oral TRE at roughly same time parenteral TRE was decreased During first 48 h parenteral TRE not decreased by more than 30 ng/kg/min Q24H and oral TRE not increased by more than 6 mg TDD Q24H	Successful transitions: 32 (17–48) mg TDD Not Successful Transitions: 35 (12–48) mg TDD	13/24 Transitions successful Transition appeared successful in nearly all patients with enrollment PVR 4.2 WU Unsuccessful transition patients (*n* = 11) experienced deterioration in FC and 10/11 experienced worsening hemodynamics 9/11 required transition back to parenteral TRE; 2/11 required additional oral therapy
Jarrett (2018) [38]	Retrospective cohort study, *n* = 14	FC: I–III, ERA: 5 PDE5-I:10 sGCS: 1 Combination:4	47 (15–75) ng/kg/min, 3 years (0–10)	Inpatient	Method not reported, 3 (1–5) days	27 (9.75–60) mg TDD	14/14 Transitions successful, hemodynamics and FC remained unchanged following transition
Ali (2019) [37]	Case series, *n* = 9	Not reported	Not reported, 13.57 months	Inpatient	Administered 1/3 TDD oral TRE, Parenteral TRE decreased by 33% 4 h later. Pattern alternated for 3 doses and 3 rate reductions. Parenteral TRE turned off 4 h after third dose, 24 h	26 mg TDD	9/9 Transitions successful Average 6MWD increased 84 m by 3 months
Coons (2016) [10]	Case series, *n* = 7	FC: II–III, Most receiving PDE5-I ± ERA	42 ng/kg/min (24–70 ng/kg/min) median (range), > 30 days	Inpatient	Oral TRE initiated at 0.5–1 mg TID and titrated in increments of 0.5–1 mg TID. Parenteral TRE down-titrated with each increase in oral TRE dose. 4 days of overlap required, not reported	8 (2.5–13) mg TID median (range)	5/7 Transitions successful 2/7 patients required transition back to parenteral TRE (AEs and worsening *n* = 1; AEs *n* = 1)
Suliman (2018) [36]	Retrospective cohort study, *n* = 6	FC: I–II, ERA + PDE5-I	Median:52.5 ng/kg/min Range: 30–64 ng/kg/min, not reported	Inpatient	Simultaneous daily up-titration of oral TRE and down-titration of parenteral TRE median: 4.5 (3–7) days	6–12 mg TID	6/6 Transitions successful One patient experienced worsening dyspnea and hemodynamics that required additional titration of oral TRE as an outpatient
Bryan (2017) [35]	Retrospective cohort study, *n* = 6	Not reported	15–52 ng/kg/min, not reported	Inpatient	Parenteral TRE dose decreased every 6–12 h, total transition time: 24–72 h	3.4–8.6 mg TID	6/6 Transitions successful No significant changes in hemodynamics following transition; no readmission or significant adverse effects following transition
Ackerbauer (2017) [34]	Case series, *n* = 2	FC: II, PDE5-I	74 ng/kg/min, not reported 80 mg/kg/min, 2.5 years	Outpatient	Parenteral TRE dose decreased by 5–12 ng/kg/min every 24–72 h, not reported	14 mg TID 11 mg TID	½ Transitions successful Patient admitted with worsening right heart failure and transitioned back to parenteral therapy after 2 months
Smith (2018) [33]	Case series, *n* = 2	FC: I–II, ERA + PDE5-I	29 ng/kg/min 22.5 ng/kg/min, Not reported	Inpatient	Parenteral TRE dose reduced with an equivalent oral TRE dose increase every 12 h, 7 days/9 days	8 mg BID 6 mg BID	2/2 Transitions successful 6MWD, BNP, and echocardiography remained stable at follow-up
Fearon-Clarke (2018) [32]	Case report, *n* = 1	FC: II, Not reported	40 ng/kg/min, not reported	Outpatient	Parenteral TRE decreased by 6 ng/kg/min per week while oral TRE initiated and titrated by 1 mg TID, 7 weeks	7 mg TID	Transition successful, Oral TRE titrated further after exertional dyspnea and fluid retention Following titration to 10 mg TID patient stabilized
Felder (2017) [31]	Case report, *n* = 1	FC: III, PDE5-I + ERA	60 ng/kg/min, 20 months	Outpatient	Parenteral TRE decreased by 4–5 ng/kg/min every 3 days. Following down-titration to 35 ng/kg/min, oral TRE 2.5 mg TID initiated. Oral TRE titrated by 0.5–1 mg TID every 2–3 days, 40 days	10 mg TID	Transition unsuccessful, 6 months after transition, patient was transitioned back to parenteral TRE following dose limiting side effects (nausea) and worsening PAH symptoms
Sargent (2020) [26]	Case report, *n* = 1	FC: II, PDE5-I	47 ng/kg/min, 6 months	Inpatient	Parenteral TRE decreased upon oral TRE initiation. Oral TRE increased and Parenteral TRE decreased every 8 h, 3 days	9 mg TID	Transition unsuccessful, After several months patient was transitioned back to parenteral TRE following deterioration to FC IV and hospitalization
Gleason (2015) [30]	Case report, *n* = 1	FC: III–IV, PDE5-I + ERA	42 ng/kg/min, 5 days	Inpatient	Parenteral TRE decreased by 50% 1 h following initiation of 2 mg TID of oral TRE. Oral TRE dose doubled after 3 doses while parenteral TRE dose was reduced by 50% until discontinued, 4 days	8 mg TID	Transition successful, Patient reported improved exercise capacity

*6MWD* 6-min walk distance, *AE* adverse effects, *BID* twice daily, *BNP* B-type natriuretic peptide, *CO* cardiac output, *ERA* endothelin receptor antagonist, *FC* functional class, *mg* milligram, *mmHg* millimeters of mercury, *mPAP* mean pulmonary arterial pressure, *ng/kg/min* nanogram/kg/min, *NP* not provided, *PAH* pulmonary arterial hypertension, *PDE5-I* phosphodiesterase type 5 inhibitor, *PVR* pulmonary vascular resistance, *Q24H* every 24 h, *QID* four times daily, *sGC* soluble guanylyl cyclase stimulator, *TID* three times daily, *TDD* total daily dose, *TRE* treprostinil, *WU* wood units, *h* hours

Notably, two prospective, controlled cohort studies have evaluated the conversion from parenteral to oral treprostinil, making the transition between these formulations the only well-characterized, clinically validated transition method [14, 41]. Chakinala and colleagues conducted a single-arm prospective study of 33 patients assessing the outcomes of patients transitioning from parenteral to oral treprostinil. Patients enrolled in this study had been receiving parenteral treprostinil for a median duration of 3.5 years and were considered eligible if they were deemed low risk per the 2015 European Respiratory Society/European Society of Cardiology guidelines for the treatment of PAH. Subjects were initiated on oral treprostinil while their parenteral treprostinil dose was simultaneously reduced. During the first 48 h, parenteral treprostinil could be reduced by a maximum of 30 ng/kg/min while oral treprostinil could be increased by a maximum total daily dose of 6 mg. Transitions were performed in the inpatient setting over a period of 5 days. Of the 33 patients enrolled, 31 remained on oral treprostinil at 24 weeks at a median total daily dose of 44 mg (range 15–75 mg). Follow-up 6MWD, echocardiography, hemodynamics, quality of life, and patient satisfaction remained unchanged at 24 weeks except for mixed venous oxygenation, which decreased from a median of 71–68% (*p* < 0.001). The authors concluded that parenteral treprostinil patients deemed to be low risk may be candidates for transition to oral treprostinil [14]. Following these results, there have been several published reports of successful transitions using this protocol. In a subsequent retrospective analysis of patients transitioned using this protocol, Maestas and colleagues found that pulmonary vascular resistance (PVR) prior to transition was greater in patients who were unsuccessful in transitioning to oral treprostinil, and also found that a cutoff PVR of 4.16 Wood units (WU) discriminated patients with successful versus unsuccessful transitions [39].

In contrast to the study by Chakinala et al., Miller and colleagues aimed to utilize a short course of parenteral treprostinil to facilitate aggressive titration of oral treprostinil [41]. The impetus behind this prospective, multicenter study was the finding that patients treated with parenteral treprostinil and then transitioned to oral were able to achieve higher doses of oral therapy than in those patients initiated on oral treprostinil de novo [41]. In the Miller study, patients with low- to intermediate- risk PAH were initiated on parenteral treprostinil, which was titrated over 2–8 weeks to a goal dose of 20 ng/kg/min. Patients were then transitioned to oral treprostinil over a period of up to 21 days. Transitions were performed at the discretion of the investigator and could take place in the inpatient or outpatient setting. The primary endpoint was the percentage of patients achieving a total daily dose of 12 mg or greater. Of the 29 patients included, 28 were successfully transitioned and remained on oral treprostinil at 16 weeks. The mean duration of parenteral treprostinil was 55 days, and 13 patients transitioned over a median of 2 days while hospitalized, whereas 16 patients transitioned over a median of 5 days in the outpatient setting. The mean total daily treprostinil dose was 16.4 mg at 16 weeks. Improvements were seen in several clinical parameters at 16 weeks including REVEAL Lite 2 score, 6MWD, N-terminal pro-B-type natriuretic peptide, WHO FC, and right atrial area. Patients also experienced improvements in adverse effects, such as headache and vomiting, following transition [41, 42].

### Inhaled to Oral Treprostinil

Limited data exist regarding the transition from treprostinil inhalation solution to oral treprostinil in patients with PAH (Table 2). The most frequently reported reasons for transition included intolerance to treprostinil inhalation solution, patient preference, worsening PAH, and poor compliance. Transitions occurred predominantly in the outpatient setting and were carried out as both crossover transitions and direct transitions [10, 43, 44, 46, 47]. Details on transition techniques were limited, with only two publications providing specifics [10, 34]. Although a minority of patients required transition back to inhaled treprostinil, discontinued treprostinil post-transition, or were escalated to parenteral therapy, most patients remained on therapy and continued to increase the dose of oral treprostinil without experiencing serious adverse events [10, 33, 34, 43, 44]. A Delphi study suggested that transitions from inhaled to oral treprostinil be considered in stable and lower-risk patients, or those experiencing clinical worsening and for whom parenteral treprostinil is not an option [45].

**Table 2:** Transitions from inhaled to oral treprostinil

Reference, year	Study design, sample size	NYHA FC, background therapy	Dose prior to transition, duration of therapy	Setting of transition	Transition method, duration	Post-transition oral TRE dose	Outcome
D’Albini (2017) [43]	Retrospective pharmacy claims analysis, *n* = 275; 86 chosen for chart review	FC: I–IV, Not reported	< 9 INH QID: 11 9 INH QID: 55 > 9 INH QID: 20, 588 ± 484 days	Not reported	Crossover: *n* = 50; direct transition *n* = 32; unknown *n* = 4, 28 days (2–112)	Not reported	70% Transitions successful at 24 weeks 27% experienced transition failure (returned to inhaled TRE, d/c PCY therapy, death, started parenteral prostacyclin) at 24 weeks
Zwicke (2021) [44]	Retrospective cohort study, *n* = 29	FC: I–III, ERA: 21% PDE5-I/sGCS: 46% ERA + PDE5-I/sGCS: 32%	11.8 (8–18) INH QID, 643 (322–991) days	Outpatient: 79% Inpatient: 21%	Direct transition: 66% Crossover: 34%, 24 (1–57) days	3.6 mg (0.3–6.6) TDD range	Follow-up outcome assessment varied across sites FC data available for 76% of patients; 59% no change, 32% improved Persistence rates: 16 weeks: 86% 24 weeks: 79% 52 weeks: 65%
Ackerbauer (2017) [34]	Case series, *n* = 2	FC: II PDE5-I	12 INH QID 9 INH QID, not reported	Outpatient	Inhaled TRE decreased by 3 INH Q24H. Oral TRE initiated at 0.125 and 0.25 mg TID and up-titrated by 0.125–0.25 mg every 24 h, not reported	3 mg TID	2/2 Transitions successful 6MWD improved and FC stable at follow-up
Coons (2016) [10]	Case series, *n* = 2	FC: II–III, Not reported	12 INH QID, not reported	Inpatient	Inhaled TRE transitioned to parenteral TRE, Oral TRE initiated at 0.5–1 mg TID and titrated in increments of 0.5–1 mg TID. Parenteral TRE down-titrated with each increase in oral TRE dose. 4 days of overlap required, not reported	2.5 mg TID 4 mg TID	2/2 Transitions successful
Smith (2018) [33]	Case report, *n* = 1	FC: II, PDE5-I + ERA	9 INH QID, not reported	Inpatient	Crossover, 2 days	1 mg BID	Transition successful Background therapies adjusted at follow-up due to not meeting treatment goals

*6MWD* 6-min walk distance, *AE* adverse effects, *BID* twice daily, *BNP* B-type natriuretic peptide, *CI* cardiac index, *ERA* endothelin receptor antagonist, *FC* functional class, *mg* milligram, *mmHg* millimeters of mercury, *INH* inhalations, *mPAP* mean pulmonary arterial pressure, *ng/kg/min* nanogram/kg/min, *NP* not provided, *PAH* pulmonary arterial hypertension, *PDE5-I* phosphodiesterase type 5 inhibitor, *PVR* pulmonary vascular resistance, *Q24H* every 24 h, *QID* four times daily, *SOB* shortness of breath, *sGC* soluble guanylyl cyclase stimulator, *TID* three times daily, *TDD* total daily dose, *TRE* treprostinil, *WU* wood units, *h* hour

## Transitions to Inhaled Treprostinil

### Parenteral to Inhaled Treprostinil

One case report, five case series, two retrospective cohort studies, and one prospective cohort study included data regarding the transition of a total of 39 patients with PAH from parenteral to inhaled treprostinil solution (Table 3). Among these data, WHO FC ranged from I to III, with most patients receiving at least one additional PAH therapy at the time of transition. In most cases, doses of parenteral treprostinil ranged from 20 to 80 ng/kg/min, with the majority of patients receiving therapy for several years prior to transitioning to inhaled treprostinil. Several studies required patients to display signs of clinical stability prior to transitioning [47, 48].

**Table 3:** Transitions from parenteral treprostinil to inhaled treprostinil

Author, year, (Reference)	Study design, sample size	NYHA FC, background therapy	Dose prior to transition, duration of therapy	Setting of transition	Transition method, buration	Post-transition inhaled TRE dose	Outcome
De Jesus Perez (2012) [46]	Retrospective cohort study, *n* = 15	FC: II–III, PDE-5i: 4 ERA: 2 PDE-5I + ERA: 7 PDE-5I or ERA + CCB: 2	73 ng/kg/min, 113 months	Inpatient and outpatient	Inhaled TRE initiated at 3 INH and titrated daily to a goal of 9 INH QID. Parenteral TRE simultaneously weaned, not reported	Not reported	13/15 Transitions successful BNP and 6MWD stable; trend toward worsening of FC and CI 2/15 required additional oral therapy 2/15 required transition back to parenteral therapy
Kimmig (2019) [49]	Retrospective cohort study, *n* = 11	FC: I–III, Majority on PDE5-I and ERA	IV: 80 ng/kg/min SQ: 76.5 ng/kg/min, ~ 8 years	Outpatient	Parenteral TRE decreased by 10–15 ng/kg/min every 1–2 weeks INH TRE titrated at the same time intervals to 9 INH QID, 56 (34.75–67.75) days	9.625 INH QID	11/11 Transitions successful BNP, 6MWD, FC, and hemodynamics stable at follow-up
Moya-Carmona (2018) [50]	Case series, *n* = 2	Not reported	Not reported	Inpatient	Parenteral TRE reduced by 35% on day 1 and 70% on day 2 Inh Tre titrated to 9 INH QID on day 2, 2 days	9 INH QID	2/2 Transitions successful Follow up 6MWD unchanged
Oudiz (2016) [47]	Prospective cohort study, *n* = 2	FC: II, None: 1 ERA: 1	24 ng/kg/min 33 months 14 ng/kg/min 123 months	Inpatient	Parenteral TRE initially down-titrated by 10–15 ng/kg/min with further down-titrations of ~ 5 ng/kg/min every 5–6 h Inhaled TRE titrated with each down-titration of parenteral TRE to goal of 6 INH minimum, 24–36 h	9 INH QID	2/2 Transitions successful Post-transition hemodynamics acceptable, 6MWD, BNP stable at follow-up
Enderby (2014) [48]	Case series, *n* = 2	FC: I–II, PDE5-I + ERA: 1 PDE5-I + ERA + CCB: 1	59 ng/kg/min 24 months 65 ng/kg/min 54 months	Inpatient	Prior to transition parenteral TRE decreased to 25 ng/kg/min or less Parenteral TRE decreased by 33%, 2 h later inhaled TRE initiated at 3 INH every 4 h, process repeated until goal dose of 9 INH achieved, 3 days	9 INH QID	2/2 Transitions successful, hemodynamics, FC, and 6MWD stable ½ required further titration of inhaled TRE due to SOB and functional changes
Raina (2013) [51]	Case series, *n* = 2	FC II, PDE5-I + ERA	38 ng/kg/min, 24 months 61 ng/kg/min, 96 months	Inpatient	Inhaled TRE initiated at 3 INH and titrated to goal of 9 INH QID Parenteral TRE decreased by 5 ng/kg/min every 6 h, 3–4 days	9 INH QID	2/2 Transitions successful, hemodynamics, FC, and 6MWD stable
Traiger (2011) [52]	Case series, *n* = 2	FC: III, None: 1 PDE5-%:1	48 ng/kg/min, > 10 years (off therapy 1 month prior) 60 ng/kg/min, > 10 years	Outpatient	Parenteral TRE decreased to 12 ng/kg/min over 2 months Inhaled TRE then initiated at 3 INH QID and titrated weekly to goal dose of 9 INH QID	9 INH QID	2/2 Transitions successful, FC and 6MWD stable
Singh (2010) [53]	Case series, *n* = 2 FC: II	FC: II, PDE5-I:1 PDE5-I + ERA: 1	46 ng/kg/min, 54 months 50 ng/kg/min, 72 months	Inpatient	Inhaled TRE initiated at 3 INH QID and titrated to goal dose of 9 INH QID. Parenteral TRE decreased by 25% daily, 4 days	9 INH QID	2/2 Transitions successful, post-transition hemodynamics stable ½ experienced worsening 6MWD and hemodynamics requiring dose escalation at 5 months ½ experienced worsening 6MWD and dyspnea at 8 months; transition to parenteral TRE under consideration
Ataya (2016) [54]	Case report, *n* = 1	FC: II, PDE5-I + ERA	Not reported, 32 months	Outpatient	Inhaled TRE initiated at 3 INH QID and titrated to goal dose of 9 INH QID. Parenteral TRE decreased every 6 h, not reported	Not reported	Transition successful, FC and 6MWD stable at follow-up

*6MWD* 6-min walk distance, *AE* adverse effects, *BID* twice daily, *BNP* B-type Natriuretic Peptide, *CI* cardiac index, *ERA* endothelin receptor antagonist, *FC* functional class, *mg* milligram, *mmHg* millimeters of mercury, *INH*: inhalations, *mPAP* mean pulmonary arterial pressure, *ng/kg/min* nanogram/kg/min, *NP* not provided, *PAH* pulmonary arterial hypertension, *PDE5-I* phosphodiesterase type 5 inhibitor, *PVR* pulmonary vascular resistance, *Q24H*: every 24 h, *QID* four times daily, *SOB* shortness of breath, *sGC* soluble guanylyl cyclase stimulator, *TID* three times daily, *TDD* total daily dose, *TRE* treprostinil, *WU* wood units, *h* hours

The predominant strategy was a crossover approach where treprostinil inhalation solution was initiated and titrated to a goal dose of 9–12 breaths (QID), while parenteral treprostinil was simultaneously weaned off [47–54]. A goal of 9 breaths was likely chosen based on clinical study data, as there is no established dosing equivalency for parenteral and inhaled treprostinil as is the case with oral treprostinil [16]. The transition setting was split between inpatient and outpatient settings; however, details on the duration of transition were limited. Overall, 37/39 transitions were deemed successful, and patients remained on inhaled treprostinil without treatment-limiting adverse effects or hospital readmissions [47–54]. Two patients from one of the retrospective analyses required reinitiation of parenteral therapy due to worsening PAH symptoms [47].

### Oral to Inhaled Treprostinil

Transition from oral to inhaled treprostinil was evaluated in one retrospective analysis (WHO FC II–IV patients) [55]. Reasons for transition included adverse events, disease progression (*n* = 12) or physician/patient preference (*n* = 3). Transition strategies included cross-titration (*n* = 6), abrupt discontinuation of oral treprostinil the day prior to initiation of inhaled treprostinil (*n* = 5), and down-titration and discontinuation of oral treprostinil prior to initiation of inhaled treprostinil (*n* = 1). Most patients underwent transition at home (*n* = 13). For the patients that were cross-titrated, the mean dose of inhaled treprostinil at the time of discontinuation of oral treprostinil was 8 breaths per session. Two patients failed transition from oral to inhaled treprostinil due to cough and death. An expert Delphi panel suggested starting inhaled treprostinil at 3 breaths QID and up-titrating to higher doses [45].

## Treprostinil Inhalation Solution to Inhalation Powder

The transition from treprostinil inhalation solution to treprostinil inhalation powder was evaluated in a Phase Ib, open-label study in 51 patients with pulmonary arterial hypertension [25, 56]. The study determined that systemic exposure from the nebulizer and dry powder were equivalent. Over the 3-week treatment phase, the majority of patients tolerated the transition well, with no treatment-related serious adverse events reported. These findings support the feasibility and clinical benefit of transitioning to inhalation powder, given ease of use, portability, and potential to improve adherence.

## Conclusion

Treprostinil is a mainstay of PH therapy, with each formulation demonstrating efficacy across a wide spectrum of patients with PAH [3, 4]. The availability of inhaled, oral, and parenteral formulations in the United States allows providers to transition between these formulations due to a change in clinical status, administration challenges, or intolerable adverse effects. However, specific guidance on how to conduct these transitions is lacking. Although the parenteral, oral, and inhaled formulations are approved by the United States Food & Drug Association, their availability outside of the United States is limited. Inhaled and oral treprostinil are not yet approved by the European Medicines Agency.

The current body of evidence describes numerous scenarios in which patients were successfully transitioned between treprostinil formulations. These transitions were often conducted in response to intolerance to parenteral therapy, adverse effects, or patient preference. Site-specific protocols often required patients to demonstrate signs of clinical stability to be eligible for transition [10, 14, 43]. Transition from parenteral to oral or inhaled prostacyclin in patients with high-risk clinical features was likely considered inappropriate, as parenteral prostacyclin therapy is the treatment of choice in this patient population. Furthermore, there is a risk of clinical deterioration following transition off parenteral therapy in this subset of high-risk patients [57]. The success of parenteral to oral or inhaled transitions can likely be attributed to careful selection of patients deemed low- to low–intermediate risk and should therefore be strongly considered when making transition decisions. In contrast, transitions to parenteral treprostinil were often performed as an escalation of therapy in response to clinical worsening or deterioration [27, 29].

In general, transitions occurred more often in the inpatient setting utilizing a crossover approach, where the prior dosage form is down-titrated and the new formulation is up-titrated to balance the risk of over/under-dosing, efficacy/ineffectiveness, and increased adverse effects. Crossover transition timelines varied from 24 h to months, with a majority taking place over a week or less. Successful transitions occurred in roughly 80% of cases reviewed; however, it should be noted there was no standardized definition for success across publications.

These data are not without limitations. Notably, most studies are limited to case reports and retrospective analyses, which are associated with inherent bias. Additionally, patient characteristics and background therapy varied widely between each study. In the absence of established guidelines for dose equivalency between formulations, there was substantial variability in doses and transition methods chosen, with some studies choosing to transition using the crossover transition method and others using an abrupt start/stop approach. Furthermore, there was a lack of consistency of clinical parameters assessed in each publication. Finally, the definition of a successful transition varied across publications; some studies determined success by the length of time that patients maintained on the therapy to which they transitioned, while others determined success by improvements in 6MWD, PVR, WHO FC, and/or BNP.

In conclusion, multiple methods of performing transitions between treprostinil formulations have demonstrated success. Given this variability, it is recommended that healthcare providers consider a patient’s individual clinical needs when devising a transition plan. While many transitions are warranted due to a change in patient’s clinical status or due to patient preference, some are conducted due to an intolerance in therapy. The versatility allowed by different formulations of treprostinil assures that intolerance or failure of one formulation does not constitute a failure of treprostinil altogether.

## Data Availability

Data sharing is not applicable to this article as no datasets were generated or analyzed during the current study.
